# A Case of Cystic Neutrophilic Granulomatous Mastitis in a Young Breastfeeding Woman

**DOI:** 10.7759/cureus.104603

**Published:** 2026-03-03

**Authors:** Isabella Bayramov, Daniel Glotzer

**Affiliations:** 1 Medicine, Florida State University College of Medicine, Tallahassee, USA; 2 Surgery, Cleveland Clinic Indian River Hospital, Vero Beach, USA

**Keywords:** benign breast condition, benign breast mass, breast disease, cystic neutrophilic granulomatous mastitis, ultrasound breast

## Abstract

Cystic neutrophilic granulomatous mastitis (CNGM) is a rare, benign inflammatory breast condition with an unclear etiology. Proposed theories include infection and autoimmune conditions. CNGM often mimics infectious mastitis and inflammatory breast carcinoma, making diagnosis a challenge, especially in breastfeeding patients. Here, we present the case of a 29-year-old breastfeeding woman who developed a painful, ulcerating left breast mass initially suspected to be mastitis or malignancy. Imaging revealed a mixed solid and cystic lesion, and biopsy confirmed CNGM. Her symptoms improved with cephalexin and corticosteroid therapy, and the lesion resolved over two months. This case highlights the diagnostic challenges posed by CNGM and underscores the importance of biopsy for accurate diagnosis. It further suggests that both microbial and immune-mediated processes may contribute to disease development and response to therapy. Continued research is needed to clarify the roles of infectious and autoimmune factors in the pathogenesis and how these etiologies drive the management of CNGM.

## Introduction

Granulomatous mastitis (GM) is a rare, benign inflammatory breast condition of uncertain etiology characterized by granuloma formation within the breast tissue. A histologically distinct subtype, cystic neutrophilic granulomatous mastitis (CNGM), is characterized by lipogranulomas containing central lipid vacuoles rimmed by neutrophils and epithelioid histiocytes [1]. Incidence of CNGM is highest in women of child-bearing age, often with a history of pregnancy, and typically presents with a painful, unilateral breast mass [1,2]. Associated clinical features may include erythema, edema, ulceration, an abscess, or nipple involvement.

The rarity of this condition and its similar presentation to other breast conditions, such as inflammatory breast carcinoma, creates a diagnostic challenge. Increasing evidence suggests that CNGM may arise from a combination of microbial and immune-mediated processes. Several studies have demonstrated an association between CNGM and *Corynebacterium* species, particularly *Corynebacterium kroppenstedtii*, a lipophilic organism that preferentially colonizes lipid-rich breast tissue and is difficult to culture using standard techniques [3-5]. Other reports describe response to corticosteroid therapy and other immune modulator medications, suggesting a possible autoimmune basis for the disease [1,6,7]. Additionally, associations with hyperprolactinemia, pregnancy, and lactation further suggest a multifactorial process involving hormonal, microbial, and immune mechanisms [2,8,9].

Given the lack of standardized treatment guidelines, management of CNGM remains individualized and guided by limited published data. Here, we present a case of CNGM in a 29-year-old breastfeeding woman that highlights proposed etiologies, diagnostic challenges, and therapeutic considerations relevant to this uncommon but clinically significant condition.

## Case presentation

A 29-year-old Caucasian woman presented to the general surgery clinic with a two-week history of a non-healing ulcer of the left breast. She described a palpating lesion in the outer left breast that became erythematous, ulcerated, and started draining. She was four months postpartum at presentation and was breastfeeding. She denied recent fever, nipple discharge, or systemic symptoms. Her medical history was unremarkable. Her maternal grandmother had breast cancer in her 40s.

Physical examination revealed an ulcerated, granulating lesion with an underlying palpable mass at the 3 o’clock position in the left breast. No supraclavicular, infraclavicular, or axillary lymphadenopathy was detected.

**Figure 1:**
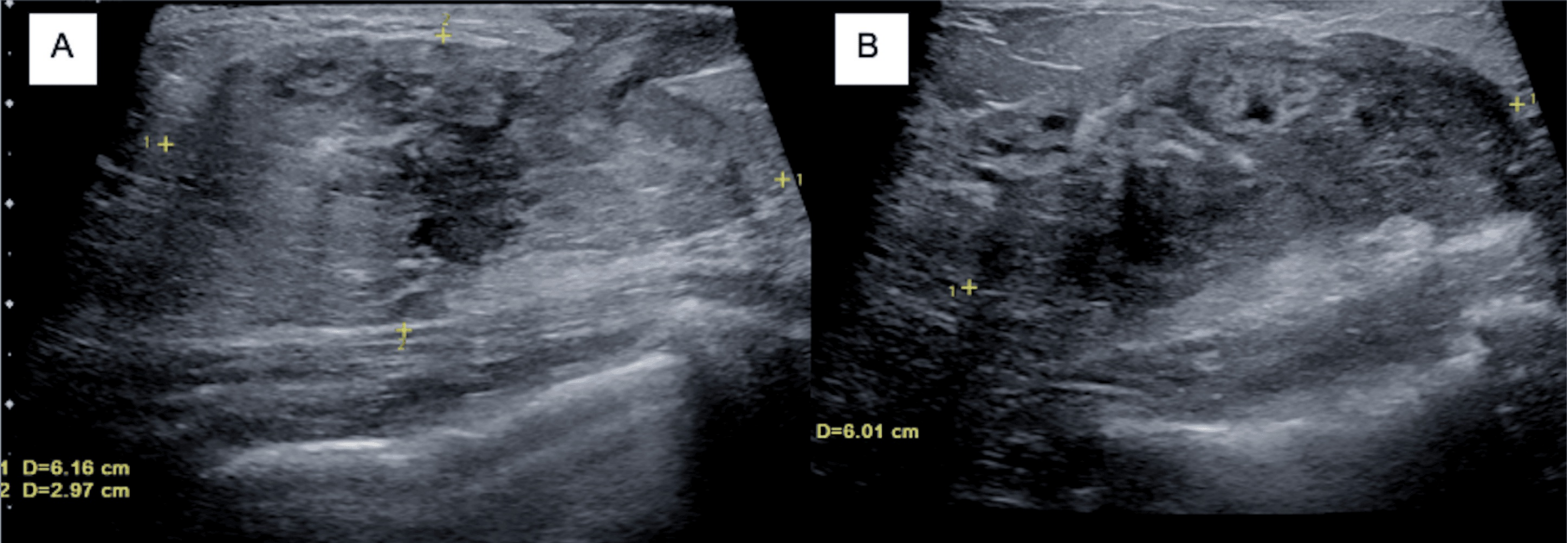
Ultrasound at initial visit: (A) antiradial and (B) radial view. Targeted ultrasound of the left breast at the area of palpable concern obtained at the patient’s initial evaluation demonstrated an ill-defined mixed solid and cystic lesion extending toward the dermis, corresponding to the site of clinical palpation.

**Figure 2:**
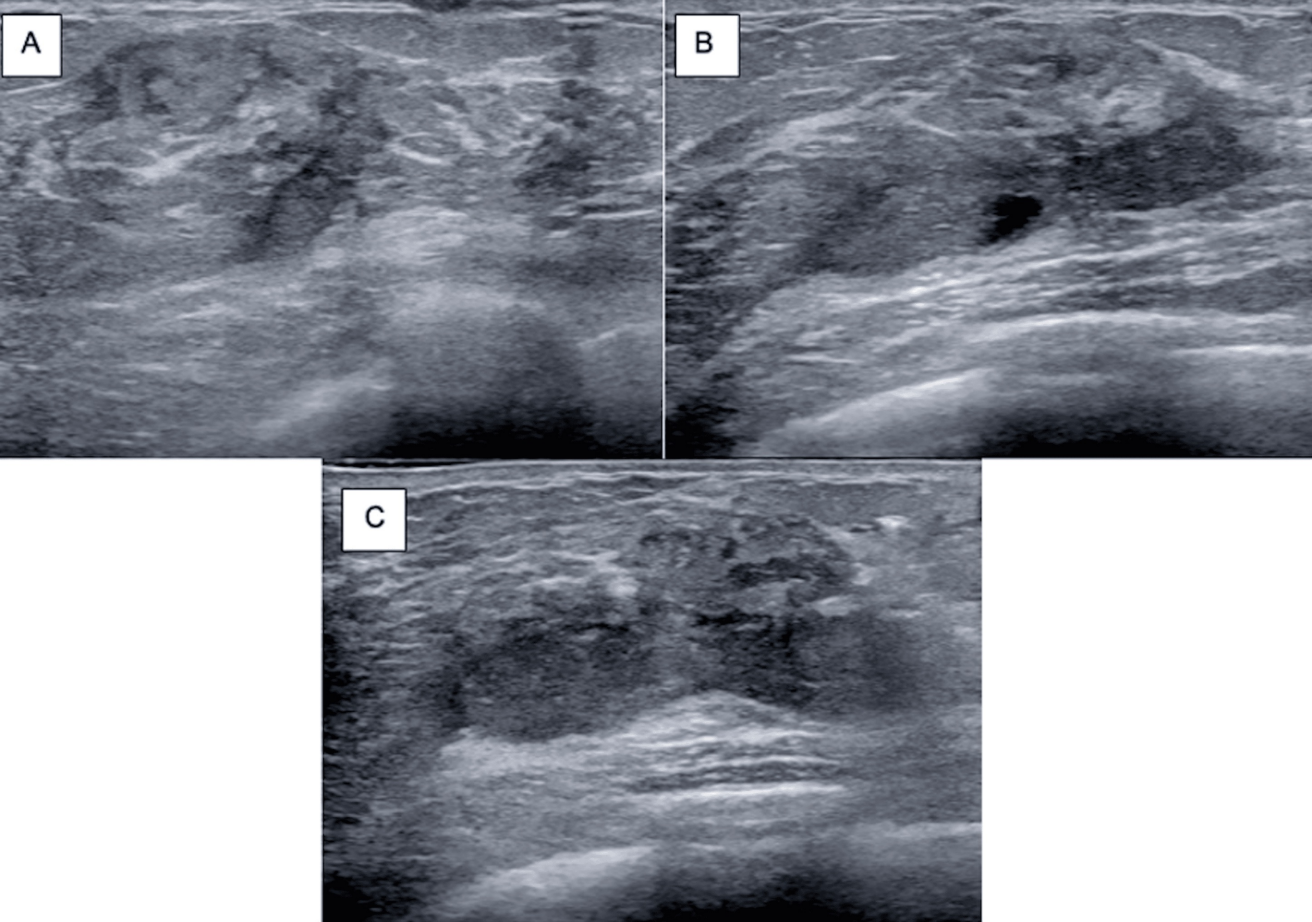
Ultrasound at the two-month follow-up: (A) antiradial, (B) radial, and (C) oblique view. Targeted ultrasound of the left breast performed at the time of ultrasound-guided biopsy, approximately two months after initiation of treatment at the initial visit, demonstrated the previously identified lesion at the biopsy site.

Ultrasound mammography of the left breast identified an ill-defined 6.6 cm solid and cystic area in the 3 o’clock position that extended to the dermis (Figure 1). An in-office fine-needle aspiration and incisional biopsy were performed at her initial visit, with cytology negative for malignant cells. Due to persistent concern for underlying pathology, she underwent an ultrasound-guided biopsy two months after initial presentation, revealing histopathologic features consistent with cystic neutrophilic granulomatous mastitis (Figure 2).

Treatment with antibiotic therapy was initiated prior to consultation with general surgery. An initial trial of doxycycline was ineffective, whereas treatment with cephalexin resulted in symptomatic improvement. Continued treatment with cephalexin was chosen, as well as initiation of a corticosteroid taper. At the two-week follow-up, she had completed all medications, the ulcer was shallower, and only local wound care was continued. The wound was closed at the two-month follow-up, and the mass was barely palpable. Rheumatologic evaluation for underlying autoimmune disease was recommended due to a possible immune-mediated component of CNGM.

## Discussion

CNGM is a unique subtype of GM, most commonly presenting in parous women of child-bearing age. Due to overlapping clinical features with more common breast conditions, CNGM is often underrecognized and frequently misdiagnosed. Literature describing cases of CNGM in actively breastfeeding patients remains limited.

The etiology of CNGM is likely multifactorial, involving microbial and autoimmune mechanisms. *C. kroppenstedtii* has been strongly implicated. Taylor et al. reviewed 34 cases of CNGM in 2003 and identified a correlation with this *Corynebacterium* species [3]. Although cultures in this case did not isolate the organism, a bacterial cause cannot be excluded. Corynebacterium species are difficult to culture, relatively rare, and often localized within the cystic spaces of the lesion [4]. In 2023, Li et al. demonstrated that in patients with isolated *C. kroppenstedtii*, bacterial growth was more prominent in lipid-rich areas of the breast, and patients had an increased likelihood of sinus tract formation, similar to the presentation observed in our patient [5].

Hormonal influences may also contribute to disease pathogenesis. Although CNGM has rarely been reported in actively breastfeeding women, associations with hyperprolactinemia, prior parity, and lactation have been described [2,8,9]. In cases reported by Kutsuna et al., two patients with hyperprolactinemia were diagnosed and treated for granulomatous mastitis in which *C. kroppenstedtii* was isolated [8]. Similarly, Wang et al. examined 62 patients with CNGM and found that 26% had elevated prolactin levels [2]. Elevated prolactin may promote inflammatory responses or facilitate bacterial growth within breast tissue. Further research is needed to clarify the role of prolactin in the development of CNGM.

The clinical course of this patient highlights the diagnostic complexity of CNGM. Presentation with a painful unilateral breast mass accompanied by erythema and ulceration raises concern for infectious mastitis with abscess or inflammatory breast carcinoma. Sarcoidosis, autoimmune conditions, and mycobacterial infection must also be considered in the differential diagnosis [10]. Although cultures were negative, early antibiotic therapy and the known difficulty in culturing *Corynebacterium *species likely contributed to these findings. Ultrasound imaging and cytology helped narrow the differential diagnosis to granulomatous mastitis and sarcoid granuloma. Ultrasound-guided biopsy was ultimately essential for histopathologic evaluation, definitive diagnosis, and differentiation of CNGM from other subtypes of granulomatous mastitis.

There are currently no established treatment guidelines for CNGM. Antibiotic therapy, particularly with lipophilic agents, has been reported to be beneficial due to the lipid-rich environment favored by *Corynebacterium* species [2,4,5,11-13]. The use of cephalexin, a more hydrophilic antibiotic, in this patient after a lack of improvement with doxycycline deviates from existing literature recommendations. This finding raises questions regarding the proposed etiologies of the disease and the relative contributions of infectious versus immune-mediated mechanisms.

Corticosteroids and other immunosuppressive therapies have demonstrated efficacy in reported cases, including treatment with dapsone and adalimumab [1,6,7]. Remission in our case following therapy that included immunosuppressive agents supports consideration of rheumatologic evaluation to reduce the risk of recurrence. Surgical excision has not been consistently shown to be effective for CNGM [1]. In this case, conservative medical management resulted in remission, and surgical intervention was not required.

This report has several limitations. It describes a single patient from a single center, which limits generalizability. Microbiologic confirmation of *C. kroppenstedtii* was not obtained, and prolactin levels were not measured. Additionally, follow-up was limited to two months, precluding assessment of long-term recurrence. Larger prospective studies are needed to better define optimal diagnostic and therapeutic strategies for this condition.

## Conclusions

This case underscores the diagnostic and therapeutic challenges of CNGM, particularly in the setting of active lactation. Awareness of CNGM as a potential diagnosis is essential when evaluating atypical or refractory breast inflammation. Tissue biopsy is critical for accurate diagnosis and to avoid unnecessary surgical intervention. The patient’s favorable response to combined antimicrobial and corticosteroid therapy supports a multifactorial disease process involving microbial, immune, and hormonal factors. However, as this report describes a single case with limited follow-up, conclusions regarding pathogenesis and optimal management should be interpreted with caution and are not broadly generalizable. Increased recognition of this entity may facilitate earlier diagnosis, appropriate management, and improved patient outcomes. Further research is needed to clarify the roles of *C. kroppenstedtii*, hyperprolactinemia, and autoimmune mechanisms in the pathogenesis and management of CNGM.
